# Role of Pre-Synaptic NMDA Receptors in the Modulation of Inhibitory Synaptic Transmission in Sensory-Motor and Visual Cortical Pyramidal Neurons in Brain Slices of Young Epileptic Mice

**DOI:** 10.21315/mjms2018.25.3.4

**Published:** 2018-06-28

**Authors:** Muhammad Hanif Che Lah, Faruque Reza, Tahamina Begum, Jafri Malin Abdullah

**Affiliations:** 1 Department of Neurosciences, School of Medical Sciences, Universiti Sains, Malaysia, 16150 Kubang Kerian, Kelantan, Malaysia; 2 Center for Neuroscience Services and Research (P3Neuro), Universiti Sains, Malaysia, 16150 Kubang Kerian, Kelantan, Malaysia

**Keywords:** pre-synaptic NMDA receptors, sensory-motor cortex, visual cortex, epileptogenesis, whole-cell patch clamp recording, brain slice electrophysiology

## Abstract

**Background:**

Previous studies from animal models have shown that pre-synaptic NMDA receptors (preNMDARs) are present in the cortex, but the role of inhibition mediated by preNMDARs during epileptogenesis remains unclear. In this study, we wanted to observe the changes in GABAergic inhibition through preNMDARs in sensory-motor and visual cortical pyramidal neurons after pilocarpine-induced status epilepticus.

**Methods:**

Using a pilocarpine-induced epileptic mouse model, sensory-motor and visual cortical slices were prepared, and the whole-cell patch clamp technique was used to record spontaneous inhibitory post-synaptic currents (sIPSCs).

**Results:**

The primary finding was that the mean amplitude of sIPSC from the sensory-motor cortex increased significantly in epileptic mice when the recording pipette contained MK-801 compared to control mice, whereas the mean sIPSC frequency was not significantly different, indicating that post-synaptic mechanisms are involved. However, there was no significant pre-synaptic inhibition through preNMDARs in the acute brain slices from pilocarpine-induced epileptic mice.

**Conclusion:**

In the acute case of epilepsy, a compensatory mechanism of post-synaptic inhibition, possibly from ambient GABA, was observed through changes in the amplitude without significant changes in the frequency of sIPSC compared to control mice. The role of preNMDAR-mediated inhibition in epileptogenesis during the chronic condition or in the juvenile stage warrants further investigation.

## Introduction

The incidence of epilepsy in children is consistently reported to be highest in the first year of life (1). In terms of age of animal models compared to human, there have been a number of efforts to correlate the age of the animal with human years (2, 3). A rat model (3) correlates with human years for the entire life span as 13.2 days in the rodent equals to one year in humans (13.2 rat days = one year human). The correlation is simply derived from the ratio of the lifespan of laboratory rats, which is an average of three years, to the worldwide life expectancy of humans, which is 80 years. This correlation means that the age of a 20–30-day-old rodent used in the current study is equal to 1–2 years in a human or an infant. However, a high concentration of extracellular glutamate, which is the main excitatory neurotransmitter and may be accompanied by alterations in the age-dependent density and/or affinity of glutamate receptors, especially of N-methyl-D-aspartate (NMDA) receptors, may lead to excessive glutamatergic transmission, assumed to be one causal factor for epilepsy (4). On occasion, the correlation of age, disease prevalence/incidence, the development of receptors in different brain regions and the interpretation of findings remain difficult. Several studies, however, have shown an increase in the density of hippocampal and cortical NMDA receptors (NMDARs) in certain animal models of epilepsy (5, 6). A principal inhibitory neurotransmitter in the neocortex, γ-amino butyric acid (GABA), has a significant role in regulating excitability during the postnatal development of the neocortex (7). GABA dictates circuit function by controlling the activity-dependent refinement of functional connections (8) and by changing the balance between excitation and inhibition (9). Plasticity at GABAergic connections has also been implicated in the establishment of synaptic networks (10).

**Figure 1:**
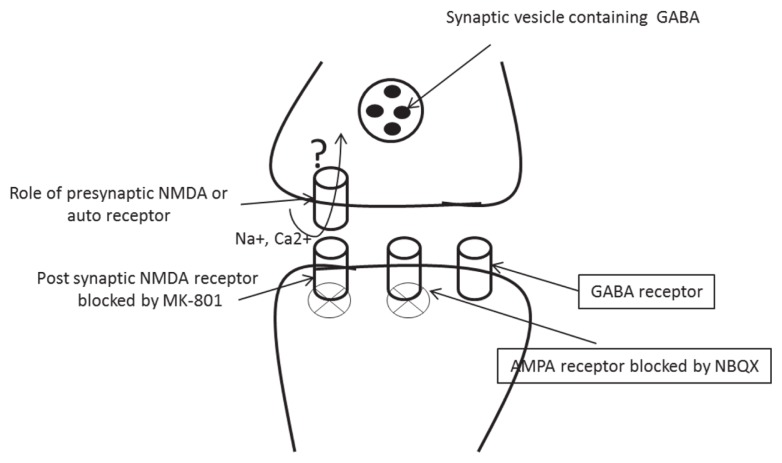
Schematic presentation of the hypothesis

NMDA receptors are traditionally viewed as being located post-synaptically at both synaptic and extra-synaptic locations; however, both anatomical (11, 12) and physiological studies (13, 14, 15, 16) have indicated the presence of pre-synaptic NMDA receptors. In the entorhinal cortex, when post-synaptic NMDARs were blocked by intracellular MK-801 (dicolzipine-an NMDA receptor antagonist), bath application of D-(−)-2-amino-5-phosphonopentanoic acid (D-APV) decreased the frequency of miniature excitatory post-synaptic currents (mEPSCs), indicating that NMDARs tonically facilitated transmitter release (15). Functional pre-synaptic NMDARs (preNMDARs) have also been reported in the visual cortex, where NMDAR subtype/subunit 2B (NR2B)-containing receptors again appeared to be involved (17). PreNMDAR expression in cortical structures is developmentally regulated (18, 19), and down-regulation has significant effects on synaptic plasticity (19). Electron-microscopic studies have demonstrated the presence of preNMDARs on GABAergic nerve terminals in the cerebellum and neocortex (20, 21). The activation of preNMDARs increases the frequency of spontaneous inhibitory post-synaptic currents (sIPSCs) and miniature IPSCs (mIPSCs) in the cerebellar basket, stellate and Purkinje cells (22, 23). Long-term potentiation (LTP) of GABAergic synapses via a pre-synaptic mechanism has been reported at synapses in the cerebellum (24) and ventral tegmental area (25). Information on the potential role of preNMDARs in inhibitory synapses or GABAergic terminals of cortical neurons is lacking. Therefore, this study was addressed about there is any involvement of pre-synaptic NMDA receptor in modulation of inhibitory synaptic transmission in sensory-motor and visual cortical pyramidal neuron in young epileptic mice, and this research question was thematically presented in Figure 1.

## Materials and Methods

### Animals

All experiments were performed in accordance to a protocol approved by the Animal Research Ethics Committee of Universiti Sains Malaysia [USM/Animal Ethics Approval/2010/(60)(248)], and extension of the period for Animal Ethics Approval was achieved when needed.

### Induction of Epilepsy

All drugs, except scopolamine, and normal saline, were administered through intraperitoneal (i.p.) injection. The weight of the mouse was measured using a balance (Precisa) for the calculation of the volumes of scopolamine methylenitrate (dose of 1 mg/kg) and pilocarpine chloride (dose of 320 mg/kg) solutions needed for each injection. Scopolamine was injected subcutaneously (s.c.) 30 min prior to pilocarpine in order to reduce the effect of systemic cholinergic activation as described previously (26). Salivation and excretion by the mouse were examples of the effects of systemic cholinergic activation. After the injection of pilocarpine i.p., the mouse was observed for the development of behavioural status epilepticus (SE), as indicated by the presence of stage 4/5 seizures within 10 min. The behavioural seizure stages were based on a previous report (27). The Racine stages were i) mouth and facial movement, ii) head nodding, iii) forelimb clonus, iv) rearing with forelimb clonus and v) rearing and falling with forelimb clonus, where stage ‘v)’ represented full motor seizure.

### Telemetric Electroencephalography (EEG) Recording

**Figure 2:**
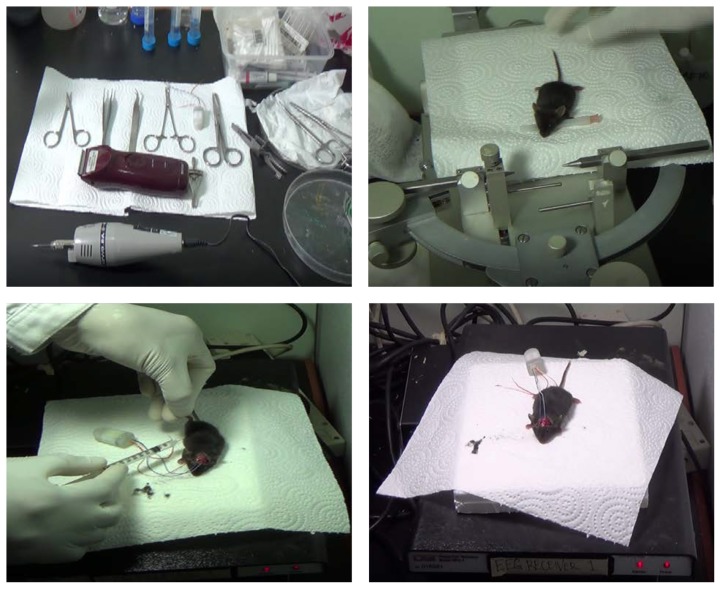
Telemetric EEG recording. Upper left panel showed surgical apparatus. Right upper panel depicted the stereotactic frame to insert intra-cortical electrode at proper place. Lower left panel showed pilocarpine injection to induce epilepsy and lower right panel showed receiver for EEG recording

To observe the development and confirm the occurrence of acute pilocarpine-induced epilepsy, in addition to behavioural status epilepticus, Telemetric electroencephalography (EEG) recording was conducted on a mouse anaesthetised with a ketamine and xylazine cocktail (80 mg/kg and 7.5 mg/kg, respectively), and additional doses were given when there was a sensorial pain reaction after pinching the footpad of the mouse during stereotaxic surgery (28). EEG was recorded from the motor cortex after implantation of the electrodes through two holes that were drilled bilateral to bregma, and another electrode was placed in the neck muscle for electromyography (EMG) artefacts. The Small Animal CNS Telemetry acquisition system (DSI) was used for recording EEG during seizure induction and first captured a five-minute stable baseline EEG waveform. After that step, 320 mg/kg of pilocarpine chloride solution was injected into the mouse (i.p.) without an interruption in the recording. The procedure was described briefly in the Figure 2. At the end of the experiment, the unconscious mouse was euthanised by decapitation and properly disposed.

### Preparation and Solution

Male C57BL/6J mice (aged 20–30 day) were deeply anaesthetised with chloroform before the whole brain was extracted from the skull and immersed in ice-cold carbogenated (95% O_2_, 5% CO_2_) artificial cerebrospinal fluid (ACSF) at 290 to 320 mOsm/L, containing (in mM): 126 NaCl, 2.5 KCl, 2 CaCl_2_, 2 MgSO_4_, 1.25 NaH_2_PO_4_, 26 NaHCO_3_, 10 D-glucose (26).

### Slice Preparation

Coronal slices of sensory-motor and visual cortices (350 μm thick) were prepared as described by Yoshimura, et al. (29), with modifications, using a vibratome (HM 650V; Microm, Germany) and incubated for 1 h in an ACSF recovery chamber perfused with carbogen at 33 °C.

### Recording

In a recording chamber a slice was perfused with carbogenated ACSF containing a bath application of non–NMDA glutamate receptor antagonist 2,3-dihydroxy-6-nitro-7-sulfamoyl-benzo(f) quinoxaline (NBQX) (50 μM) to block pre- and post-synaptic α-amino-3-hydroxy-5-methyl-4-isoxazolepropionic acid (AMPA) receptors. The whole-cell patch clamp technique was performed at a holding potential of +15 mV (V_hold_ *= +*15 mV*)* on neurons at layer V of the sensory-motor cortex and layer IV of the visual cortex to record sIPSCs using a computer-controlled microelectrode amplifier, Axon Multiclamp 700B (Molecular Devices, USA) and low-noise data acquisition system, Axon Digidata 1440A (Molecular Devices, USA). The K-based intracellular solution (ICS) containing (in mM) 135 KCl, 10 HEPES, 2 Na-ATP, 0.2 Na-GTP, 2 MgCl_2_, and 0.1 EGTA was used for recording, and 1 mM of MK-801-based intracellular solution was introduced to the neuron to block post-synaptic NMDARs using the recording glass micropipette, with a resistance of 3–5 MΩ. Traditionally, NMDA receptors were blocked by magnesium (Mg^2+^), and in order to unblock the receptors, action potential-dependent sIPSCs were recorded without Tetrodotoxin (TTX), a sodium (Na) channel blocker. To avoid multi-synaptic inhibition, evoked responses from pre-synaptic stimulation were not used.

### Statistical Analysis

All data are expressed as the mean ± standard error of the mean (SEM). Two-tailed student’s *t*-tests were used to evaluate the significance of the mean differences of amplitude, frequency, rise time and decay time of the sIPSC from the pyramidal neurons of the sensory-motor and visual cortices. The critical level of significance was set at *P* ≤ 0.05.

## Results

Regarding seizure induction, in the absence of any movement from the mouse, the baseline EEG recording was obtained before the injection of the pilocarpine. The recording did not show any epileptic EEG discharges (Figure 3A). Five to ten minutes after injection of the pilocarpine chloride solution at a dose of 320 mg/kg, generalised epileptiform EEG activities were recorded from the mouse, which were characterised by increased amplitude and high frequency paroxysmal discharges, as shown in Figure 3B. The epileptic EEG discharges were tonic-clonic with a frequency of 3–6 Hz, and the amplitude of the irregular spikes was approximately 0.025 mV.

**Figure 4:**
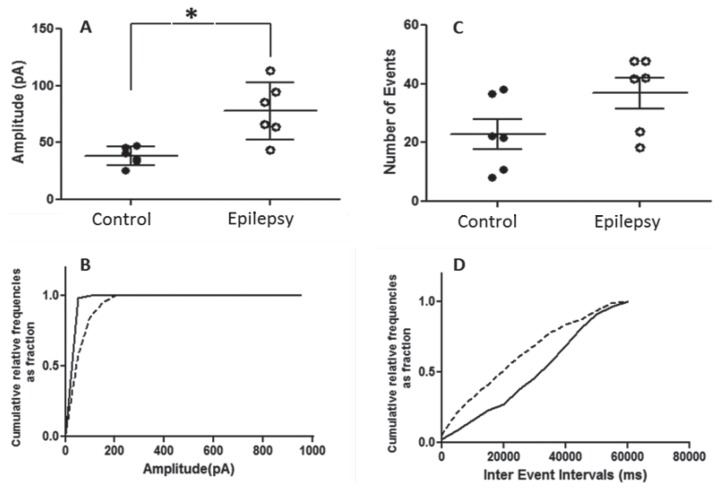
Quantitative analysis of the spontaneous inhibitory postsynaptic current (sIPSC) amplitude (pA) recorded from the neurons of the sensory-motor cortex with the pipette containing MK-801. (A) Scatter dot plot of the sIPSC amplitude (mean ± SEM, *n* = 6) from control and epileptic mice. (B) Cumulative distributions of the amplitude of six combined neurons from control (solid line) and epileptic mice (dotted line). (C) Scatter dot plot of the sIPSC frequency (mean ± SEM, *n* = 6) from control and epileptic mice. (D) Cumulative distributions of the frequency from six combined neurons of control (solid line) and epileptic mice (dotted line). *Significant, *P =* 0.026

**Figure 5:**
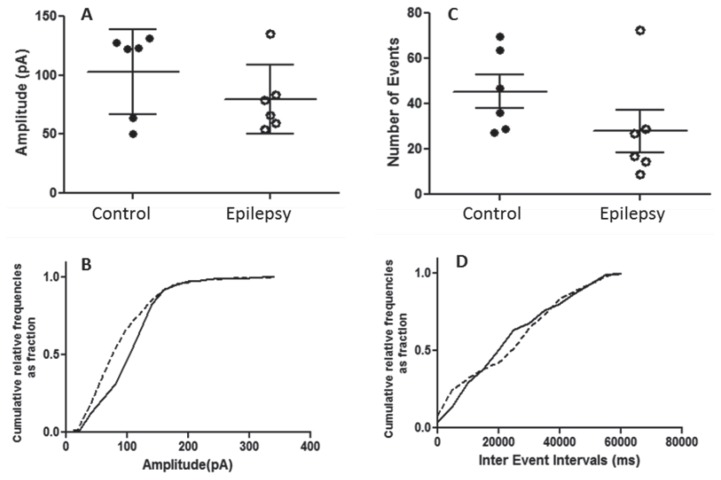
Quantitative analysis of the spontaneous inhibitory post-synaptic current (sIPSC) amplitude (pA) recorded from the neurons of the visual cortex with the pipette containing MK-801. (A) Scatter dot plot of the sIPSC amplitude (Mean with SEM, *n* = 6) from control and epileptic mice. (B) Cumulative distributions of the amplitude from six combined neurons of control (solid line) and epileptic mice (dotted line). (C) Scatter dot plot of the sIPSC frequency (mean ± SEM, *n* = 6) from control and epileptic mice. (D) Cumulative distributions of the frequency from six combined neurons of control (solid line) and epileptic mice (dotted line)

Regarding the patch clamp recording from the sensory-motor cortex, the mean amplitude of the sIPSC was found to be significantly increased (*t* = 3.120, df = 5, *P =* 0.026) in epileptic mice (77.86 ± 10.17 pA) compared to control mice (38.25 ± 3.39 pA) (Figures 4A and 4B). The mean frequency of the sIPSCs was not significantly different between the control and epileptic groups (Figures 4C and 4D). Recording from the visual cortex, the mean amplitudes (Figures 5A and 5B) and frequencies (Figures 5C and 5D) were not observed to be significantly different in the control and epilepsy groups.

**Figure 6:**
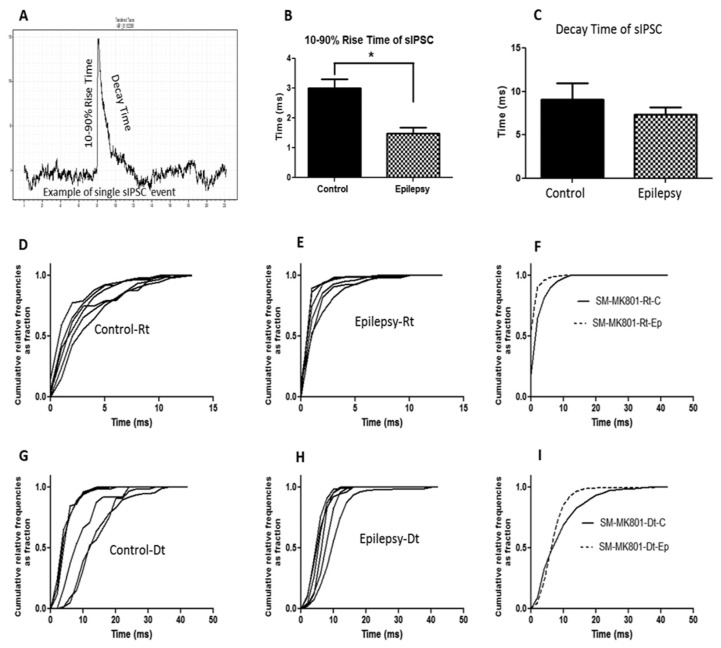
Quantitative analysis of the spontaneous inhibitory postsynaptic current (sIPSC) kinetics recorded from the neurons of the sensory-motor cortex with the pipette containing MK-801. (A) Example of an individual sIPSC showing 10%–90% rise time (ms) and decay time (ms). (B, C) Bar graph showing an average 10%–90% rise time and decay time in the sIPSC taken from 432 events for both control and epileptic mice. Data are presented as the mean ± SEM. The 10%–90% rise time (ms) of the sIPSC in epileptic mice decreased significantly. (D, E) Cumulative distributions for the 10%–90% rise time are presented from six individual neurons of control and epileptic mice. (F) Cumulative distributions of the 10%–90% rise time from six combined neurons of control (solid line) and epileptic mice (dotted line). (G, H) Cumulative distributions of the decay time are presented from six individual neurons of control and epileptic mice. (I) Cumulative distributions of the decay time from six combined neurons of control (solid line) and epileptic mice (dotted line) Rt = rise time; Dt = decay time; SM = sensory-motor; C = control; Ep = epileptic

**Figure 7:**
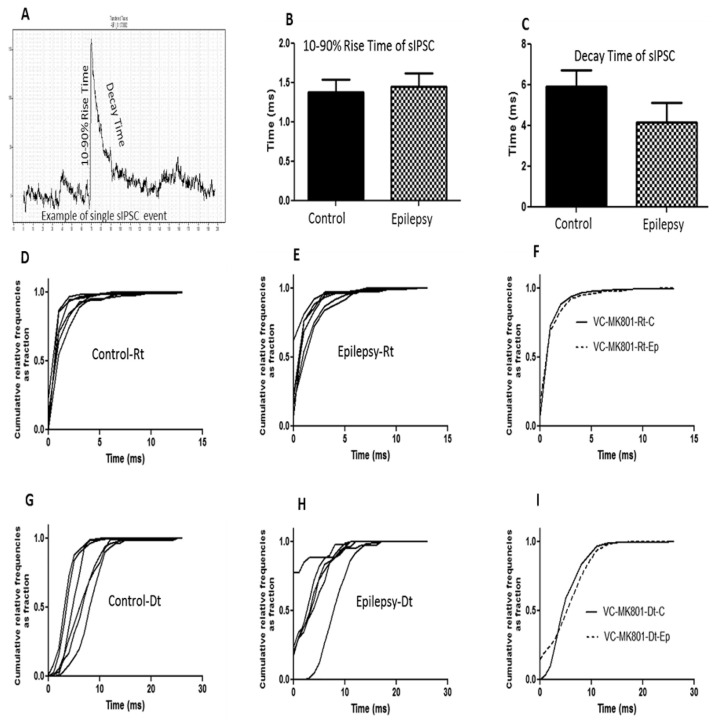
Quantitative analysis of the spontaneous inhibitory postsynaptic current (sIPSC) kinetics recorded from the neurons of the visual cortex with the pipette containing MK-801. (A) Example of an individual sIPSC showing the 10%–90% rise time (ms) and decay time (ms). (B, C) Bar graph showing the average 10%–90% rise time and decay time of sIPSC taken from 773 and 504 events for control and epileptic mice, respectively. The data are presented as the mean ± SEM. The 10%–90% rise time (ms) of the sIPSC in epileptic mice decreased significantly. (D, E) Cumulative distributions of the 10%–90% rise time are presented from six individual neurons of control and epileptic mice. (F) Cumulative distributions of the 10%–90% rise time from six combined neurons of control (solid line) and epileptic mice (dotted line). (G, H) Cumulative distributions of the decay time are presented from six individual neurons of control and epileptic mice. (I) Cumulative distributions of the decay time from six combined neurons of control (solid line) and epileptic mice (dotted line) Rt = rise time; Dt = decay time; VC = visual cortex; C = control; Ep = epileptic

Regarding the rise time and decay time of the sIPSC (Figures 6A–6I and 7A–7I) there were no significant differences found in the decay time among the control and epilepsy groups in both the sensory-motor and visual cortical areas. There were, however, significant differences (*t* = 4.207, df = 5, *P =* 0.008) in the mean sIPSC 10%–90% rise times between the control (3.01 ± 0.29 ms) and epileptic (1.49 ± 0.19 ms) groups in the sensory-motor cortical area.

## Discussion

The aim of this study was to record the amplitude, frequency and kinetic properties of spontaneous inhibitory post-synaptic currents (sIPSCs) in the sensory-motor and visual cortices of the mouse model in the inhibitory synapses of pyramidal neurons to discover the role of the pre-synaptic NMDA receptor (preNMDAR) in epilepsy. The result showed that the sIPSC recorded from epileptic young mice increased in amplitude without remarkable changes in the frequency compared to control mice, suggesting a post-synaptic mechanism. The hypothesis was that the synaptic transmission of GABA might be changed in an underlying mechanism of epileptogenesis through pre-synaptic NMDA receptors mediated by calcium (Ca^2+^) influx in young mice.

By name, pre-synaptic receptors are located at pre-synaptic locations on a neuron. Therefore, preNMDAR influences the efficacy of synaptic transmission by increasing and decreasing neurotransmitter releases (30). Not only excitatory, but inhibitory/GABAergic synapses also possess preNMDARs in the cerebellum and neocortex (20, 21). Increased frequency of spontaneous and miniature inhibitory post-synaptic currents (mIPSCs) is directly related to the activation of preNMDARs in the cerebellar area, which has been well recognised (22, 23, 31). A previous study proved that preNMDARs are present at specific synapses (32), but not available at all synapses or in all brain areas with all ages; moreover, preNMDAR decreases over development. The expression of the preNMDAR occurs more in younger than older animals. Functional preNMDARs were observed in excitatory synapses/neurons of the hippocampus [postnatal day (PND) < 5] in a previous study (33), in the entorhinal cortex (PND = up to five weeks) (15), in the primary visual cortex (PND = up to three weeks) (16), and in the frontal cortex (PND = up to 20 days) in inhibitory synapses (34). The presence of preNMDAR in inhibitory synapses of the sensory-motor and visual cortical areas is not well-established. In the first postnatal weeks, the activity of preNMDAR gradually decreased in the primary visual cortex in a previous study (35). Similarly, our study proved that the expression of preNMDAR in the sensory-motor and visual cortical areas appeared less or absent at PND 20–30 in inhibitory synapses, as sIPSC frequency did not significantly change in our study, which can be explained from the abovementioned and from other previous studies (15, 16, 33, 34). The minimal current in NMDARs was enough to depolarise the nerve endings to open the Mg^2+^ block in post-synaptic neurons (34), and the activation of post-synaptic NMDARs influenced the facilitation of the amplitudes of sIPSCs in the inhibitory synapses in the brain areas. Similarly, this phenomena is applicable for the pre-synaptic NMDA receptor, and we assumed that the opening of the preNMDAR by relieving the Mg^2+^ block with the minimal current would allow cation influx, especially the Ca^2+^, which may influence the synaptic transmission of GABA release; however, we did not observe that in this study, as the increased amplitudes of the sIPSC in the sensory-motor areas were instead due to transient up-regulation or alteration of the post-synaptic GABAR (36). Absent or reduced firing of action potentials can prevent neurotransmitter release in the post-synaptic neuron. Less frequency means the pre-synaptic neuron is not activated adequately (16). The peak synaptic conductance in epileptic mice was slower compared to control, as demonstrated in the differences in the rise time. The visual cortical areas did not show any differences in the kinetic properties of the sIPSC, which may indicate different receptor types in the different cortical areas. Interestingly, the visual areas did not show significant changes in any properties of the sIPSC in the epileptic condition but rather showed a slight opposite result from the sensory-motor cortex. A previous study (37) showed that pilocarpine-induced status epilepticus occurred due to the excitotoxic effects of vascular, ischaemic or hypoxic damage of the neuron, which might involve different mechanisms because of the different vasculature in different brain areas. In addition, the composition of the preNMDAR is highly diverse, and the activation or expression of the preNMDA receptor can either increase or decrease the synaptic activity depending on its cellular type and distribution in brain areas (38). Finally, the mean amplitudes, frequencies, rise time and decay time in the epileptic group compared to the control group proved that the preNMDAR has no significant role in the modulation of inhibitory synapses during epilepsy at acute stages in young mice in our study. The mechanism of the modulation of inhibitory synapses during epilepsy might follow different pathways, which warrants further investigation.

## Conclusions

The results of this study indicate that the pre-synaptic inhibition mediated by the pre-synaptic NMDA receptor in acute brain slices in pilocarpine-induced epileptic mice of young age was not significantly manifested. In an acute case of epilepsy, a compensatory mechanism of post-synaptic inhibition may be mediated by ambient GABA, as indicated by increased amplitude and no significant changes in frequency of the sIPSC compared to control mice. The role of preNMDAR-mediated inhibition in the epileptogenesis of the chronic condition needs to be investigated further in term of preNMDAR involvement in the modulation of inhibitory synaptic transmission in neocortical pyramidal neurons in juvenile non-human vertebrates.

## Figures and Tables

**Figure 3A:**
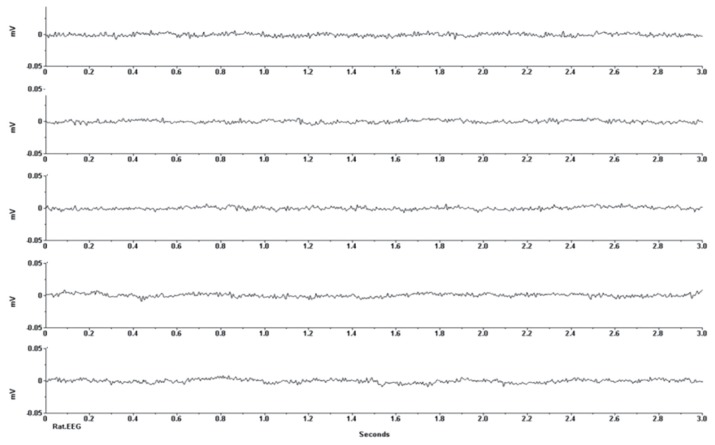
Continuous EEG recordings from the sensory-motor cortex of an anaesthetised mouse before the injection of the pilocarpine chloride solution

**Figure 3B:**
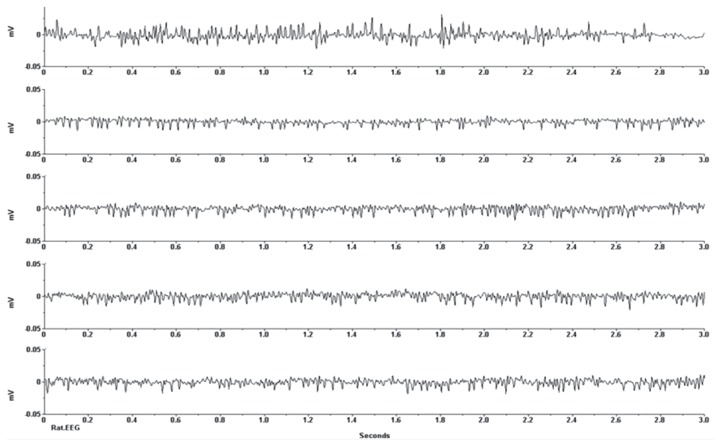
Continuous EEG recordings from the sensory-motor cortex of the anaesthetised mice showing a generalised spike 10 min after the injection of 320 mg/kg of pilocarpine chloride solution
